# USP10 as a Potential Therapeutic Target in Human Cancers

**DOI:** 10.3390/genes13050831

**Published:** 2022-05-06

**Authors:** Li Tao, Xiao Liu, Xinya Jiang, Kun Zhang, Yijing Wang, Xiumin Li, Shulong Jiang, Tao Han

**Affiliations:** 1 The Affiliated Cancer Hospital of Zhengzhou University, Zhengzhou 450008, China; nbtl137@163.com; 2 School of Basic Medical Sciences, Xinxiang Medical University, Xinxiang 453003, China; liuxiao1210xiaoliu@163.com (X.L.); jxy15255706987@163.com (X.J.); z1106405586@163.com (K.Z.); wyj60939@163.com (Y.W.); 3 Henan Key Laboratory of Tumor Molecular Therapy Medicine, Xinxiang Medical University, Xinxiang 453003, China; lxm3029981@126.com; 4 Clinical Medical Laboratory Center, Jining First People’s Hospital, Jining Medical University, Jining 272000, China

**Keywords:** deubiquitinating enzymes (DUBs), USP10, tumorigenesis, inhibitors, therapeutic strategy

## Abstract

Deubiquitination is a major form of post-translational protein modification involved in the regulation of protein homeostasis and various cellular processes. Deubiquitinating enzymes (DUBs), comprising about five subfamily members, are key players in deubiquitination. USP10 is a USP-family DUB featuring the classic USP domain, which performs deubiquitination. Emerging evidence has demonstrated that USP10 is a double-edged sword in human cancers. However, the precise molecular mechanisms underlying its different effects in tumorigenesis remain elusive. A possible reason is dependence on the cell context. In this review, we summarize the downstream substrates and upstream regulators of USP10 as well as its dual role as an oncogene and tumor suppressor in various human cancers. Furthermore, we summarize multiple pharmacological USP10 inhibitors, including small-molecule inhibitors, such as spautin-1, and traditional Chinese medicines. Taken together, the development of specific and efficient USP10 inhibitors based on USP10’s oncogenic role and for different cancer types could be a promising therapeutic strategy.

## 1. Introduction

Dysfunctional protein expression is associated with many diseases including cancer. Proteins, especially in eukaryotic cells, maintain normal cell function under steady-state conditions, 80% of which is mediated by the ubiquitin–proteasome system (UPS) [1]. Ubiquitin is an 8.5 kDa protein containing 76 amino acids that produces a polyubiquitin chain on a target protein, mainly through its seven lysines at the N-terminal and methionine sites. Ubiquitin usually binds to a target protein through glycine at the C-terminal and leads to its degradation through the 26S proteasome system [2,3]. In this process, three kinds of ubiquitin enzymes play a significant role: E1 ubiquitin-activating enzyme, E2 ubiquitin-conjugating enzyme, and E3 ubiquitin ligase. E3 ubiquitin ligase is target-specific and regulates the interaction of ubiquitin with target proteins. More details about the structure and function of UPS have been well summarized elsewhere [4].

Deubiquitination is a process that is the opposite of ubiquitination, and deubiquitination enzymes (DUBs) play an important role in this process. DUBs remove or cleave the isopeptides between the substrate protein and ubiquitin to achieve deubiquitination. This process of reversal had not been widely studied until now [5,6]. Although some researchers have found that DUBs affect a variety of cellular processes, their underlying mechanisms, targets, and inhibitors for specific diseases remain largely unknown [7]. The regulation of DUBs and the abundance of their downstream target proteins is considered a novel and promising cancer-treatment strategy [8].

Although hundreds of DUBs have been identified, the characteristics and functions of many in human diseases remain unclear [9]. USP10 has been widely studied and found to be involved in various cellular processes, including DNA repair, cell-cycle regulation [10], autophagy [11], and immune and inflammatory responses. Especially for inflammation, Li et al. demonstrated that USP10 can elevate proinflammatory factors in endometriosis (EM) and that Cai’s Neiyi Prescription (CNYP) can reduce EM-induced inflammation by inhibiting the mRNA and protein expression of USP10 [12]. However, it is known that USP10 maintains normal cell function by controlling the protein balance through the ubiquitin–proteasome degradation pathway [13]. In immune system diseases, such as asthma and systemic sclerosis, USP10 influences inflammatory responses by inhibiting T-box transcription factor (T-bet) ubiquitination and stabilizing its expression, and thus, it has also been found that quercetin, an inhibitor of USP10, alleviates asthma via enhancing the ubiquitination and promoting the degradation of T-bet [14]. Other studies have also demonstrated that USP10 deubiquitinates AMPKα, regulating energy metabolism [15], and stabilizes the CD36 protein, thus promoting the development of atherosclerosis [16].

In recent years, mounting evidence has also indicated that USP10 plays a crucial role in tumorigenesis [9,17,18,19,20,21,22,23]. The overexpression of USP10 can inhibit the formation of stress granules (SGs), thus restraining the development of a tumor [19,24]. In addition, two well-known tumor suppressors, P53 and SIRT6, can be regulated and considered as substrates of USP10 [25,26]. The p53 protein is a key transcription factor and plays an important role in the DNA-damage response, cell-cycle arrest, and apoptosis, which are closely associated with tumorigenesis. Under normal conditions, the E3 ubiquitin ligase MDM2 maintains a basal low level of p53 by promoting its ubiquitination and 26S proteasomal degradation. In 2020, Yuan et al. revealed that USP10 is a novel regulator of p53. The depletion of USP10 significantly reduced p53’s stabilization by increasing its ubiquitination. Moreover, the expression of p53’s downstream target genes, including p21 and Bax, was also downregulated [27]. Mechanistically, USP10 can reverse the activity of Mdm2 through deubiquitinating p53 in the cytoplasm, causing the return of p53 from the cytoplasm to the nucleus and affecting the nuclear output of the p53 protein. Moreover, USP10 plays an essential role in stabilizing the p53 protein in neurodegenerative diseases, and several proteins regulate the cell cycle by regulating USP10, affecting the stability of p53. For example, miR-191, a factor promoting the proliferation of pancreatic cancer cells, acts by negatively regulating USP10 and thereby reducing the content of p53 [28]. In thyroid cancer and gastric cancer, DZNep, an essential component of PRC2, also regulates p53 by regulating the content of USP10 [29,30]. Interestingly, a negative-feedback loop between USP10 and p53 was observed by Luo et al., who found that miR-138 could decrease the activity of USP10 through specifically binding to the 3′-UTR of USP10’s mRNA following an increase in the expression of the p53 protein, but in turn, p53 reduced the expression of miR-138, forming a feedback pathway [31]. It is worth noting that, in addition to regulating DNA damage via p53 ubiquitination, USP10 also stabilizes MSH2 (an important DNA mismatch-repair protein) and TRAF6 (an activator of NF-κB), which affects DNA repair and other physiological processes during DNA damage [23,25,27,32,33]. In line with this, some studies found that the disruption of the interaction between USP10 and p53 inhibited cancer cells’ viability or tumor growth. For example, mycoplasma DnaK particles inhibited the anticancer activity of p53 through binding to USP10 [34]. The inhibition of the proline hydroxylase PHD3 could significantly reduce the binding of USP10 to p53 [35]. Resveratrol promotes the p53-mediated apoptosis of cancer cells by acting on the USP10-binding protein G3BP1 [36]. IGF2BP3 (insulin-like growth factor 2 mRNA binding protein 3) inhibits p53 expression through disrupting the interaction between USP10 and p53 and promotes lung cancer progression [37]. Although some substrates and regulators of USP10 have been reported, the role of USP10 in tumorigenesis remains poorly understood. Herein, we focus on the involvement of USP10, along with its substrates and upstream regulators, in cancer cells. We expect that the summary of such knowledge will help us to develop new inhibitors or strategies to target USP10 in human cancers in the future.

## 2. USP10 Is a USP-Family Deubiquitinating Ligase

The USP family is a relatively large group (with more than 50 known members) of deubiquitinating enzymes [38,39]. As the largest and most diverse family of DUBs, the USPs have many similarities with and differences from other DUBs, such as OTUs (ovarian tumor-related proteases), JAMMs (Jad1/Pad/MPN domain-containing metalloenzymes), UCHs (ubiquitin C-terminal hydrolases), MJDs/Josephin (Machado–Joseph disease proteases), and the ZUP1/ZUFSP (zinc finger with UFM1-specific peptidase) family. All these DUBs can reverse ubiquitination by cleaving the peptide or isopeptide bond and removing ubiquitin from its substrates. However, they can be differentially classified into cysteine proteases and metalloproteases. For example, the JAMMs are known to be metalloproteases, but the OTUs, USPs, MJDs/Josephin, and UCHs are considered cysteine proteases [40]. Interestingly, among the cysteine-protease DUBs, the USPs are larger than the other DUBs [9]. More importantly, they consist of one specific USP domain (also known as the catalytic domain) and three other conserved subdomains, which form a hand-like structure including a thumb, finger, and palm [41]. Different domains or subdomains play important roles in regulating the enzyme activity.

All the USPs contain short motifs (two short conserved fragments, a lysine box, and a histidine box) and a finger-like structure containing β-lamellar structures that coordinate and support ubiquitin’s ligands, mediating the interaction with the ubiquitin substrate. The thumb is composed of the central catalytic helix, nucleophilic cysteine, and core proteasome structure. The palm contains aspartic acid, histidine residues, and the conserved β-sheet structure of the protease core. Therefore, the thumb–palm gap provides the catalytic center for the USP domain [9,13,15,42]. Recent results from NMR structural studies show that the active site undergoes rearrangements upon Ub binding to a USP. Subsequently, a conformational change in the catalytic domain promotes the catalytic hydrolysis of Ub from the ubiquitinated proteins. 

**Figure 1:**
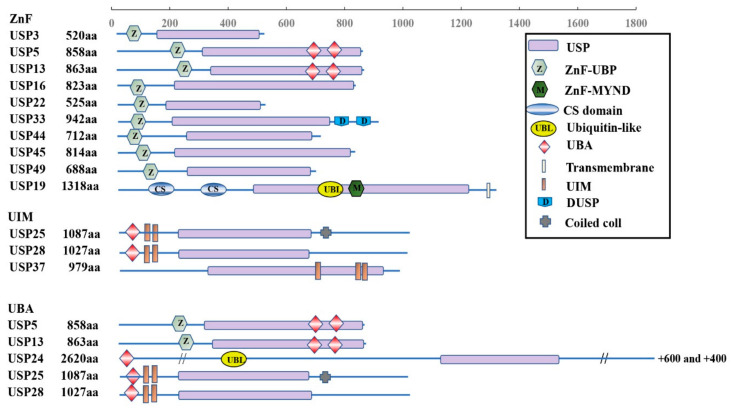
Schematic illustration of ZnF, UIM, and UBA subfamily architectures.

**Figure 2:**
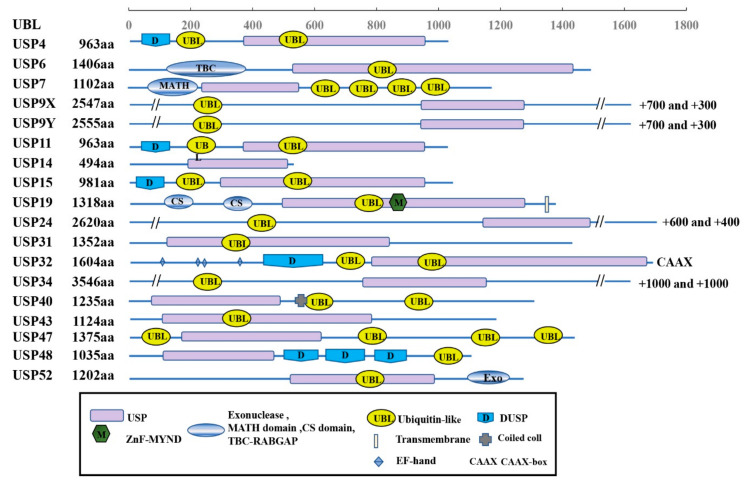
Schematic illustration of UBL-related subfamily architectures.

**Figure 3:**
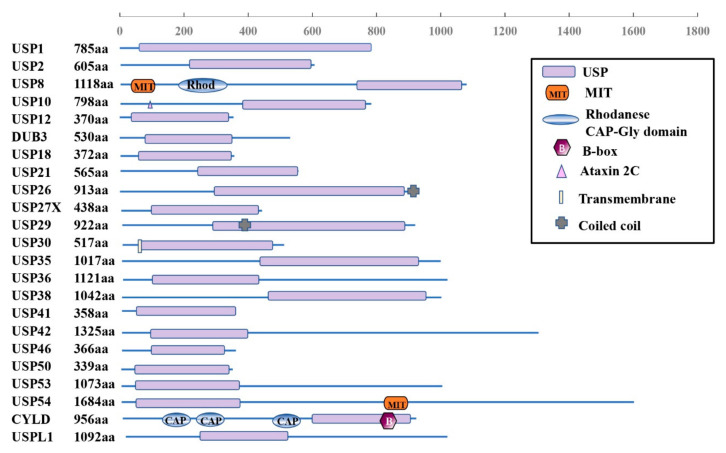
Schematic illustration of USP10-related subfamily architectures.

The USPs are grouped into five subfamilies based on the ubiquitin domain’s architecture: Ub-associated domain (UBA domain) (5 members), Ub-interacting motif (UIM) (3 members), zinc-finger Ub-specific protease domain (ZnF-UBP domain) (10 members) (Figure 1), and the common potential ubiquitin domain (UBL domain) (17 members) (Figure 2). All these members have very similar ubiquitin-related domains (different from the USP domain). However, USP10 is a member of the family only including the USP domain (about 23 members) (Figure 3) and having no UBA, UIM, ZnF-UBP, or UBL domain (Figure 1, Figure 2 and Figure 3) [9,13].

The domain structures of the ZnF, UIM, and UBA subfamilies are shown in Figure 1. There is a zinc finger Ub-specific protease domain (ZnF-UBP domain) in ten members (upper), Ub-interacting motif (UIM) in three members (middle), and Ub-associated domain (UBA domain) in five members (lower). However, USP5 and USP13 from the ZnF family, together with USP25 and USP28 from the UIM family, also have UBA domains.

The domain structures of the UBL-related subfamily are shown in Figure 2. All the members have the domain of the ubiquitin-specific proteases (USPs) and ubiquitin-like domain (UBL domain). In addition, different members have other additional domains, such as transmembrane and coiled-coil domains.

The domain structure of the USP10-related subfamily is shown in Figure 3. All the members have the domain of the ubiquitin-specific proteases (USPs). In addition, USP8 and USP54 also contain the domain of microtubule-interacting and trafficking proteins (MIT) or rhodanese (Rhod). Both USP26 and USP19 include coiled-coil domains. However, USP30 contains a transmembrane domain and CYLD includes the CAP-Gly domain (CAP) and B-Box (B).

The human USP10 gene encodes 798 amino acids forming a typical USP domain. The USP10 consists of a larger N-terminus region, a USP catalytic structure domain (about 380 amino acids, starting at 415 amino acids from the N-terminus [43]), and a smaller C-terminus region (Figure 1) [44]. The USP10 proteins are highly conserved among humans and other mammals. For example, there is about 99% percent identity between the amino acid sequences of the human and rat or mouse forms [9].

Similar to other DUBs, USP10 can deubiquitinate and cleave Ub from the C-termini of substrates through four steps. The first step is the USP binding to the ubiquitin COOH terminus via its USP domain, causing a conformational change in the catalytic domain. Then, the conserved residues of the group (Cys, His, Asp, and Asn) form a catalytic triad in a specific manner. Next, the deprotonated thiol group conducts a nucleophilic attack on the carbonyl carbon, and the active site is transferred from its initial position. Finally, the non-anchoring Ub is removed from the target proteins [9,45]. It is known that the USPs specifically cleave ubiquitin sections or process ubiquitin chains based on the substrates. For example, both USP21 and USP7 cleave K6-linked ubiquitin chains. USP7 can also cleave K11-, K33-, K48-, and K63-linked chains [9,45]. However, whether and how USP10 can also remove K11-, K48-, and K63-linked ubiquitin chains remains largely unknown. Recently, Yuan et al. demonstrated that USP10 stabilized Smad4 via directly binding to Smad4 and removing a Lys48-linked polyubiquitin chain [46]. Another previous study from Hu et al. also showed that the depletion of USP10 increased the K48-linked polyubiquitination of HDAC6 in the non-small-cell lung cancer (NSCLC) cell line H1299. In addition, He et al. discovered that USP10 reduced the K63-linked polyubiquitination of PTEN in the NSCLC A549 cell line [47]. All these modifications affect the protein’s stability when targeted by USP10.

It has been reported that many other proteins, such as TP53 RPS2, RPS3, RPS10, and LC3B, can be deubiquitinated by USP10 and are known substrates (as summarized in Table 1). Although many of their ubiquitin sites have been identified, the mechanism of ubiquitin recognition by USP10 remains elusive. It will be intriguing to assess how and which ubiquitin linkage could be recognized and cleaved by USP10 in vitro and in vivo. More importantly, the characteristics of the specificity for chains other than K48- and K63-linked ubiquitin of USP10 is also worthy of future investigation.

## 3. Regulation of USP10

**Figure 4:**
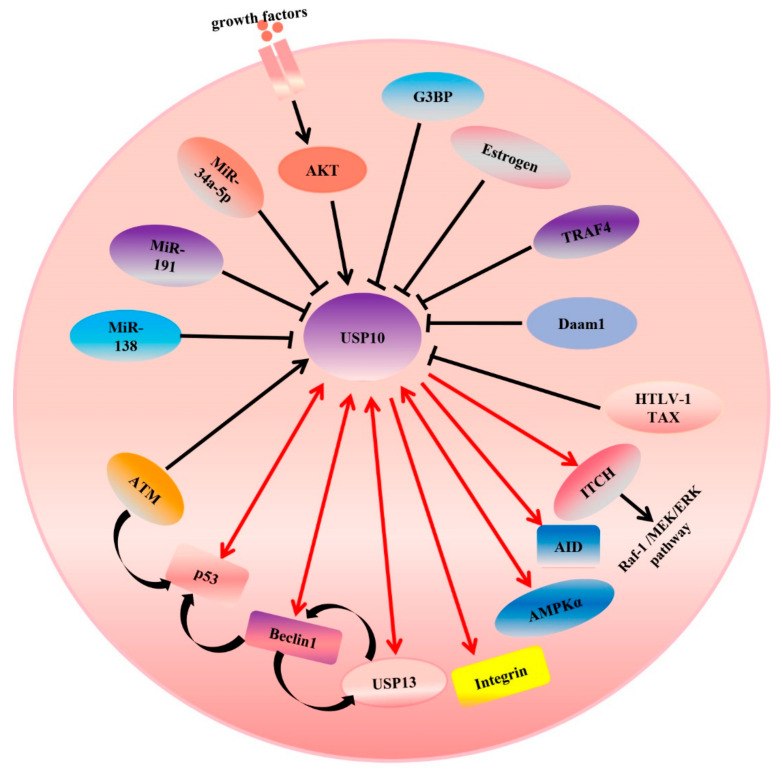
USP10 targets different signaling pathways in cancer cells.

As for most deubiquitinating enzymes, the activity of USPs can be regulated by multiple mechanisms, including those acting at the transcriptional and post-translational levels. Previous studies showed that a subset of USP-family deubiquitinating enzymes including USP1, USP4, USP8, and USP13 could be phosphorylated by CDK1, AKT, or CLK3 kinase, preventing the USPs and substrates binding [45]. For example, the CDK1-mediated serine phosphorylation of USP1 disrupts the interaction between USP1 and UAF1. CLK3 mediated the phosphorylation of USP13 at Y708, promoting its binding to c-Myc, which is an important transcription factor and also known as an oncogene in many cancers [71]. In line with the above studies, USP10’s activity can be regulated by phosphorylation at its N-terminus domain. Deng et al. revealed that AMPKα directly mediated the phosphorylation of Ser76 at the USP10 N-terminus and increased its activity. Interestingly, USP10 could regulate the deubiquitination of AMPKα, creating a positive-feedback pathway [15] (Figure 4). Yuan et al. demonstrated that ATM could phosphorylate USP10 at Thr42 and Ser337 upon the DNA-damage response and USP10 was translocated into the nucleus, where the N-terminus of USP10 (amino acids 1–100) binds to p53 and inhibits its ubiquitination [27]. In addition, Luo et al. reported that co-stimulation by BCR and TLR1/2 initiated the AKT-dependent phosphorylation of T674 of USP10; subsequently, USP10 entered the nucleus and stabilized the AID protein (also see Figure 4 and Table 2) [72]. In addition to phosphorylation directly controlling the activation of USP10, a previous study from the Deng group revealed that, in keloid cells, TRAF4 inhibited USP10-mediated p53 deubiquitination and degradation through disrupting the access of p53 to USP10 [73]. Liu et al. found that USP10 stabilized beclin-1 by interacting with and inhibiting its ubiquitination. Very interestingly, they also pointed out that beclin-1 controlled the stability of USP10 by regulating the stability of USP13, which can deubiquitinate USP10 (also see Figure 4) [53]. Another finding regarding feedback regulation was reported by several groups [33,53,74,75]. They found that MCPIP1 directly binds to USP10 and serves as a bridge between USP10 and TANK [33]. MCPIP1 forms a complex with USP10 and TANK, which mediates the deubiquitination of the TRAF6 K63 chain [76] and inhibits NF-κB activation upon DNA damage [33]. On the other hand, genotoxic-stress-induced NF-κB activation enhanced MCPIP1 transcription [75]. In addition, Lin et al. demonstrated that USP10 enhanced the stability of SIRT6 through interacting with its N-terminal regulatory domain and deubiquitinating SIRT6, and then inhibited the transcriptional activity of the c-Myc oncogene through SIRT6 [25]. Consistent with the latest findings, Zhou et al. found that USP13 also affected the ubiquitination of c-Myc and resulted in enhancing its stability [77]. However, feedback regulation between USP10 and c-Myc was also found. c-Myc directly binds to the second E-box sequence of the USP10 gene and activates the transcription of USP10 [69]. All these findings suggest that regulatory feedback mechanisms play an important role in signaling pathway regulation and maintaining a balance in protein levels. They also encourage us to identify many more modifiers of USP10, such as kinases and DUBs, and uncover the mechanisms of USP10’s dynamic regulation. This highlights that identifying more regulators of USP10 will be necessary for clarifying its role in disease. 

An arrowhead from USP10 indicates positive regulation of the substrate protein. Single arrowheads with red lines indicate that USP10 only regulates the substrates, while double arrowheads with red lines indicate that they can regulate each other. For example, USP10 regulate the activity or expression of integrin, AID, ITCH, p53, USP13, and so on. Conversely, p53 and USP13 also can regulate USP10 forming feedback loop. An arrowhead toward USP10 indicates a positive regulation of USP10’s deubiquitinating ligase activity, while a blunt head toward USP10 indicates negative regulation of such. As shown, both AKT and ATM increase the activity of USP10. However, overexpression of miR-138, miR-191 and miR-34a-5p inhibits the mRNA and protein expression levels of USP10. A curved arrowhead with a black color indicates positive regulation.

In addition to USP10 being regulated by the proteins of host cells, previous studies of the Takahashi group showed that HTLV-1 Tax interacted with amino acids 727–798 at the C-terminal of USP10 and inhibited USP10’s deubiquitination activity and function. For example, inhibiting USP10 reduces the production of reactive oxygen species and suppresses apoptosis and the formation of SGs (stress granules) [78]. On the other hand, it has been found that USP10 can be regulated at the transcriptional level. Generally, a microRNA binds to the 3-untranslated region (3′-UTR) of an mRNA in a sequentially specific manner, inducing the mRNA’s degradation or inhibiting its translation. Luo et al. revealed that USP10 is a target of miR-138 and that the 3′-UTR conserved domain of USP10 directly interacts with miR-138. The overexpression of miR-138 inhibits the mRNA and protein expression levels of USP10 (also see Figure 4 and Table 2). Moreover, they also found that p53, which can be deubiquitinated by USP10, negatively regulates the expression of miR-138 through binding to the miRNA’s promoter region [31]. In other words, there is feedback regulation. Another microRNA, miR-191, is considered an upstream regulator of USP10 in PDAC (pancreatic cancer) cells [28]. All the regulators of USP10 are summarized in Table 2.

Zhang et al. indicated that miR-34a-5p acted as a negative regulator of USP10, but the underlying mechanism of the microRNA’s action remains largely unclear [79]. More importantly, whether there is feedback regulation is also unknown. This highlights that identifying more regulators of USP10 will be essential for clarifying the mechanisms and roles in different diseases, especially for cancer.

## 4. USP10 in Cancers

USP10 plays multiple roles in many diseases including human cancers. It was shown that the overexpression of USP10 promoted the proliferation and metastasis of multiple tumors including adult T-cell leukemia, glioblastoma multiforme, chronic myeloid leukemia, non-small-cell lung cancer, hepatocellular carcinoma (HCC), colon cancers, etc., while it has also been reported that the expression of USP10 was reduced in gastric carcinoma (GC), HCC, and colon cancer and suppressed the development of tumorigenesis through regulating different signaling pathways, such as the p53 apoptosis pathway, suggesting that USP10 is, indeed, a double-edged sword in different cancer types or different cell contexts for the same cancer. We will further discuss the oncogenic and tumor-suppressor roles of USP10 in the following section.

### 4.1. USP10 as an Oncogene

It was first shown that increased expression of USP10 predicted poor survival in patients with glioblastoma multiforme (GBM) [83]. Mounting evidence has indicated that USP10 plays an oncogenic role in tumorigenesis. In adult T-cell leukemia (AML), previous studies have revealed that the human T-cell leukemia virus type 1 (HTLV-1) oncoprotein Tax interacts with USP10 and promotes ROS-dependent apoptosis and the occurrence of AML. Moreover, USP10 was also found to reduce the sensitivity of AML cells to chemotherapeutic drugs including arsenic [19,78]. Recently, the Weisberg group verified that increased expression of mutant FLT3–ITD correlated with poor prognosis and low survival. Mechanically, USP10 led to the accumulation of FLT3–ITD through deubiquitinating and stabilizing the mutant FLT3–ITD, promoting AML progression [9,61]. In addition to mutant FLT3–ITD, another spleen tyrosine kinase (SYK), a critical regulator of FLT3, was deubiquitinated and stabilized by USP10; depleting or inhibiting USP10 reduced the expression of SYK, which promoted the proliferation of Ba/F3 and MOLM14 cells [68,84].

Chronic myeloid leukemia (CML) is positively associated with abnormally high expression of the tyrosine kinase BCR-ABL. Liao et al. found that both USP10 and SKP2 are highly expressed in the primary monocytes of patients with CML than in the primary monocytes of healthy humans. Increased USP10 led to the deubiquitination and stabilization of SKP2, causing BCR-ABL activation and promoting CML cells’ proliferation [85,86]. To date, in addition to blood-related cancers, USP10 also plays oncogenic roles in many solid tumors including hepatocellular carcinoma (HCC) [46], colon cancer [68,84], renal-cell carcinoma (RCC) [27], non-small-cell lung cancer (NSCLC)[27], prostate cancer (PCa) [87,88], esophageal cancer [87,88], breast cancer [75,89], and melanoma [87,88]. In tissue samples from HCC patients, increased USP10 levels are positively correlated with the abundance of YAP/TAZ [90]. Functionally, USP10 directly deubiquitinates and stabilizes YAP/YAZ and promotes HCC progression. Additionally, other substrates of USP10 in HCC have been found, such as Smad4 and TGF-β. Yuan et al. found that USP10 directly bounds to and stabilized Smad4, promoting HCC metastasis, using a functional RNA-interference screening method; thus, USP10 is considered a prognostic and therapeutic target in Smad4-positive HCC metastatic patients [46]. In prostate cancer, high expression of USP10 indicates poor prognosis [18,91]. USP10 promotes the proliferation of PCa cell lines through binding to and increasing the stability of G3BP2, which inhibits p53 activity. On the other hand, androgen receptor (AR), a key regulator, plays a crucial role in the regulation of PCa progression. AR-related proteins including H2A, Zub1, and H2Aub1 can be deubiquitinated and stabilized by USP10. In esophageal cancer and human neuroblastoma, USP10 affects cancer cell proliferation by stabilizing PCNA and NRF-1, respectively [92]. These findings suggest that USP10 could function as an oncogene to precisely control cell proliferation in tumor cells. 

In addition to the differential effects of USP10 in different cancers, USP10 can be a double-edged sword in the same cancer. For example, in colon cancer, USP10 interacted with and stabilized SIRT6 through inhibiting SIRT6’s ubiquitination, suppressing proliferation [25]. However, the USP10-mediated deubiquitination of NLRP7 or MSI2 increased its stabilization and expression, promoting the occurrence of CRC [68,84]. It will be intriguing to assess when and how USP10 could deubiquitinate different proteins in vitro and in vivo.

In contrast to the tumor-suppressor role of USP10 in p53-WT cancer cells, USP10 exerts an oncogenic function in p53-mutant cancer cells. For example, in p53-mutant RCC cells, increased USP10 expression promotes cell proliferation via deubiquitinating and stabilizing the mutant p53 [27]. Additionally, in NSCLC, the USP10-mediated deubiquitination of the oncogenic protein histone deacetylase 6 (HDAC6) leads to cisplatin resistance in patients harboring mutant p53 [93]. In addition, USP10 affects NSCLC progression through regulating EIF4G1 in a p53-independent manner [94].

In addition to USP10 controlling cell proliferation, a study from the Ouchida group revealed that USP10 promotes tumor migration or invasion. Epithelial-to-mesenchymal transition (EMT) plays an essential role in the process of tumor invasion. USP10 facilitates tumor migration by stabilizing the protein abundance of the EMT transcription factor Slug [7]. Moreover, USP10 activates the Raf-1/MEK/ERK pathway, which is an important regulator of EMT [60]. Functionally, USP10 stabilizes the ITCH E3 ligase, enhancing the stability of MEK1 and activating the Raf-1/MEK/ERK pathway. Recently, our group reported that ITCH polyubiquitinated and activated BRAF in melanoma cells in response to proinflammatory cytokines, leading to the elevation of MEK/ERK signaling [95]. This further supports the role of USP10 in activating the Raf-1/MEK/ERK pathway. In addition, Spain-1, an inhibitor of USP10, inhibits melanoma growth and improves the anticancer effect of cisplatin by inhibiting USP10 activity [87,88]. However, the underlying mechanism remains largely unknown. 

### 4.2. USP10 as a Tumor Suppressor

To understand the role of USP10 in liver cancer, Lu et al. analyzed 74 pairs of paraffin-embedded tissues of HCC patients and adjacent non-tumor specimens (61 men and 13 women) and found that compared to low levels of USP10, high levels of USP10 predicted longer disease-free survival and overall survival. In addition, USP10’s mRNA expression was downregulated in clinical HCC tissue samples compared with adjacent non-tumor samples [96]. Mechanistically, USP10 stabilizes the expression of PTEN and AMPKα through inhibiting the Lys48-linked polyubiquitylation of PTEN and regulating the K63-linked ubiquitin chain of AMPKα; all these inhibit mTOR activation and suppress the proliferation of HCC cell lines [96]. As in HCC, USP10 was found to be downregulated in lung cancer, and the knockdown of USP10 inhibited PTEN ubiquitination and promoted tumor growth and invasion [97]. Recently, Yu et al. demonstrated that the protein and mRNA levels of the tumor suppressor KLF4 were reduced in lung cancer tissues. Moreover, the depletion of KLF4 facilitates the development of lung cancer [98]. Wang et al. further found that USP10 maintains KLF4’s stability through deubiquitinating KLF4. Similarly, MSH2 (MutS Homolog 2), another USP10 substrate, was identified in lung cancers by Zhang et al. Their results show that the depletion of USP10 in A549 increased cell survival and decreased apoptosis through destabilizing MSH2 [32]. All these results indicate that in the same cancers, USP10 affects tumor development via regulating different substrates. Growing evidence has shown that USP10 plays important tumor-suppressor roles in different cancers, such as colorectal cancer, non-small-cell lung cancer, small intestinal adenocarcinoma, epithelial ovarian cancer, colorectal cancer, renal-cell carcinoma, breast cancer, and gastric cancer [99,100,101]. In 2020, Bhattacharya summarized the partial roles of USP10 in different cancers [9]. For example, in colon cancer, USP10 interacted with and stabilized SIRT6 through suppressing SIRT6 ubiquitination [25]. Thus, increasing the expression of c-Myc and p53 further inhibits cell-cycle progression, cell growth and tumorigenesis. In addition, it has also been shown that USP10 is silenced by methylation and downregulated in the early stages of colorectal cancer [99]. Taken together, all these findings suggest that USP10 also functions as a tumor suppressor. USP10 has lower expression in GC than in para-cancer tissues. More importantly, low expression of USP10 indicates poor prognosis in GC patients, suggesting that USP10 might be a promising prognostic marker in GC [102]. Interestingly, S100A12 (also named calgranulin C) has been found to be downregulated in GC clinical samples and positively correlated with USP10 expression. Previous studies using RCC (renal-cell carcinoma) tissue microarrays indicated that USP10 expression was reduced in RCC samples compared with normal renal tissues. The reconstitution of USP10 inhibited the colony formation and cell proliferation of the CAKI-1 and CAKI-2 RCC cell lines. However, it is still unknown whether USP10 can deubiquitinate and stabilize these related proteins. Further studies need to be conducted to uncover the underlying mechanisms [102,103]. More recently, Kim et al. demonstrated that USP10 effectively suppressed curcumin-induced paraptosis in malignant breast cancer cells through a mechanism not involving the regulation of beclin-1, p53, or AMPK. All these results indicate that USP10 may also function in a deubiquitinase-independent manner [104]. Given that USP10 may be a tumor suppressor, several upstream regulators of USP10 including miR-191 and DZNep were identified in pancreatic cancer and thyroid cancer cells, separately. Mechanistically, both of them promote cell proliferation via inhibiting the expression of USP10, which further decreases the stability of p53 [29,30]. 

Overall, all the above studies suggest that USP10 can act as a tumor suppressor via different molecular and cellular mechanisms. 

## 5. Targeting USP10 in Human Cancers

Although USP10 is a double-edged sword and plays a dual role in tumorigenesis, it is widely considered a well-known oncogene in specific contexts as described above. In light of the important oncogenic role of USP10 in many cancers, inhibiting the expression or activity of USP10 is expected to be a promising therapeutic strategy for curing a range of human tumors. In line with the notion, many researchers have developed multiple screening methods to identify small-molecule inhibitors for cancer treatment, such as activity-based probes, Ub-7-amino-4-methylcoumarin(AMC), Ub-phospholipase A2 (PLA2), time-resolved fluorescence resonance energy transfer (TR-FRET), SDS-PAGE–Coomassie, and many others [105]. Different methods have different advantages and disadvantages. For example, UB-PLA2 cannot be used to screen inhibitors of the UCH family, but TR-FRET is very sensitive for screening UCH-family inhibitors. However, compared with UB-PLA2, a major problem is the lack of commercial kits available for TR-FRET methods. More details about these methods have been reviewed by Chen et al. [105]. In recent years, several inhibitors have been developed based on the structure or enzyme activity of USPs, such as GW7647 for USP1 [106], Q29 for USP2 [107], PR619 for USP2/4/5/7/8/15/20/28/47 [105], H9X19818 for USP7/10 [108], and VLX1570 for USP14 [109]. Among them, VLX1570 was approved for clinical trials as the first DUB inhibitor [109]. 

Given VLX1570’s potential to promote lung-tissue damage, caused by the accumulation of the drug’s metabolites, it has been demonstrated to lead to many adverse effects, especially severe respiratory insufficiency [105,110]. Therefore, screening and developing small-molecule inhibitors specifically targeting the USPs could be a potential and promising strategy for human cancer therapy. Below, we focus on the progress regarding USP10 inhibitors.

### 5.1. Spautin-1

In 2011, it was reported for the first time that spautin-1 was a highly potent autophagy inhibitor, as demonstrated using imaging-based screening. Spautin-1 inhibits autophagy by reducing the activity of USP10 and USP13, which promotes a major aspect of autophagy: degradation by Vps34 complexes. In addition, spautin-1 also downregulated the expression of USP10 and USP13 at the protein level [53]. Given the role of spautin-1 in autophagy, spautin-1 can improve the therapeutic efficacy of IM for CML patients. Mechanically, spautin-1 significantly inhibits imatinib mesylate (IM)-induced autophagy in CML cells by downregulating beclin-1 and enhances IM-induced apoptosis by inactivating PI3K/AKT and GSK-3β [111]. CFTR plays an important role in cystic fibrosis (CF) [112]. Although it has been shown that USP10 deubiquitinates and degrades CFTR [64], Pesce et al. found that spautin-1 could not affect the expression of CFTR, indicating that spautin-1 affected CF progression in a USP10-independent manner. However, the underlying mechanism is largely unknown. Interestingly, spautin-1 was recently shown to suppress cell growth or kill cancer cell lines in an autophagy-independent manner. A report from the Yang group revealed that spatutin-1 promoted immunogenic cell death (ICD) through the activation of the JUN transcription factor in response to mitochondrial oxidative injury, ultimately resulting in the upregulation of many cytokines including CXCL10 (C-X-C motif chemokine ligand 10) and IL-6 (interleukin-6) [113]. In addition, the inhibition of USP10 by spautin-1 significantly suppressed the migration and metastasis of HCC. Further evidence revealed that USP10 directly binds to Smad4 and stabilizes Smad4 through deubiquitination [46].

Moreover, spautin-1 suppresses the survival of ovarian cancer, prostate cancer, melanoma, and NSCLC cells in a USP10-independent manner [114]. More recently, spautin-A41, an analog of spautin-1, was developed by Elsocht et al. They found that both the autophagy-inhibiting effect and induction of microsomal stability were much better than those of spautin-1 [115]. However, there is still no evidence showing that spautin-1 or spautin-A41 directly binds to USP10. Further investigation is obviously warranted to determine whether spautin-1 and spautin-A41 could be used in clinical trials for cancer therapy in vivo.

### 5.2. P22077 and HBX19818 (or Analogs)

Similar to spautin-1, another two DUB inhibitors were found in 2001. Altun et al. found that PR-619 and P22077 inhibited DUB activity using an activity-based chemical proteomics screening method. They further validated that PR-619 inhibited a broad range of DUBs, but P22077 only targeted USP7. Almost simultaneously, another USP7 inhibitor, HBX19818, and its analogs were developed using biochemical assays and an activity-based protein-profiling strategy. It has been demonstrated that P22077 inhibits the proliferation of neuroblastoma, colon cancer, ovarian cancer, and lung cancer cells via different mechanisms including the induction of p53-mediated apoptosis [116,117,118]. Recently, P22077 was reported to inhibit the growth and metastasis of melanoma by activating the ATM/ATR signaling pathway [119]. Notably, P22077, HBX19818, and HBX19818 analogs including Compounds 3, 7, and 9 also inhibit USP10 in mutant-FLT3-expressing cells. Among the HBX19818 analogs, Compounds 3 and 9 are more specific for USP10 but not USP7. Furthermore, Compound 3 also has a stronger inhibitory activity at low concentrations than the others. In addition, the results indicate that the IC50 of HBX19818 for USP10 is 14 μM and that of P22077 is 6 μM in these cells. Additionally, the inhibitory activity is lower than that for USP7. Importantly, both of them can directly bind to and inhibit USP10. As expected, P22077, HBX19818, and its analogs inhibited the growth of acute myeloid leukemia harboring an FLT3 mutation [61]. On the other hand, it has been reported that USP10 is closely related to the DNA-damage response. It would be intriguing to explore whether a synergistic therapy combining USP10 and an effector of DNA damage would be a more efficient strategy for cancer treatment.

### 5.3. Wu-5

Wu-5, a novel USP10 inhibitor, was found by screening an in-house compound library, and it was shown to overcome FLT3-inhibitor resistance and enhance the anti-AML effect of crenolanib. Mechanistically, Wu-5 inhibits USP10 activity through interacting with USP10 in cells. Subsequently, it reduces the expression of the downstream effector AMPKα, suppressing the growth of MV4-11 cells [120]. However, further in-depth research is required to determine whether Wu-5 has broad-range effects and specificity in human cancer cells and clinical efficacy.

### 5.4. Quercetin

Quercetin (C15H10O7) is a pentahydroxyflavone, widely present in many fruits and vegetables. It exerts many effects combating different diseases, such as cancer, immunity diseases, and cardiovascular diseases. Dysregulation of the T-box transcriptional factor T-bet can cause many immune-mediated diseases [121,122]. A previous study by Pan et al. revealed that quercetin reduced the expression of USP10, which interacts with and maintains the level of T-bet, resulting in T-bet downregulation [14]. Therefore, quercetin was considered a T-bet inhibitor. Many studies have demonstrated that quercetin inhibits the growth of many cancer cells including those of breast cancer, colon cancer, lung cancer, and other cancers [122]. Although several clinical trials have been published, there is still no direct clinical evidence showing that it has any therapeutic effects in human cancers. Therefore, it is necessary to further clarify the role of quercetin and explore whether the inhibition of USP10 is the major mechanism for cancer treatment. It will also be interesting to screen many more flavonoids and develop quercetin derivatives targeting USP10 for anticancer therapy.

### 5.5. Traditional Chinese Medicine

Traditional Chinese Medicine plays a crucial role in the treatment of many diseases including cancer. In 2009, a medicine called Cai’s Neiyi Prescription (CNYP) was invented by Cai; it inhibits inflammation via inducing the apoptosis of endometrial stromal cells. Furthermore, they found that CNYP reduces USP10’s mRNA and protein expression. Altogether, it has been demonstrated that CNYP is an inhibitor of USP10, indicating that CNYP is a promising anticancer drug [12]. Given that Traditional Chinese Medicine has relatively few adverse effects, it will also be intriguing to test whether CNYP inhibits cancer cells’ proliferation or metastasis in vitro and in vivo, with the potential for its earlier use in patients.

### 5.6. UbV.10.1

Different from other USP10 inhibitors, UbV.10.1 is a mixture of proteins or peptides that have high affinity for USP10 and inhibit its activity. It was identified by screening a phage-displayed ubiquitin variant (UbV) library and considered an inhibitor of endogenous USP10 in cells. The overexpression of UbV.10.1 facilitates p53’s export from the nucleus to the cytoplasm and degradation through the inhibition of USP10 [123]. However, it is still unknown whether it can inhibit cancer cells’ proliferation or metastasis, etc. Additional in-depth investigation is necessary before using this inhibitor for clinical trials.

### 5.7. DZNep

3-deazaneplanocin A (DZNep) was developed based on the activity of S-adenosylhomocysteine hydrolase. It exerts anticancer effects in many cancers including blood and solid cancers through inhibiting EZH2 or other methyltransferase activity [124]. A previous report revealed that DZNEP suppressed the proliferation of TP53-wild-type cells but not TP53-mutant-type cells. One reason is that it can activate the p53 pathway by increasing USP10 expression, but it is more toxic to the majority of cancer cell lines; it has not been approved by the Food and Drug Administration (FDA). In 2020, another EZH2 inhibitor, tazemetostat, was approved by the FDA for metastatic epithelioid sarcoma treatment [125]. It is encouraging us to explore whether tazemetostat or other EZH2 inhibitors cure cancers in an USP10-independent manner. It also very interesting to test whether tazemetostat will be more efficient together with other pathway drugs such as P53, etc.

## 6. Conclusions and Perspectives

Since its discovery in 2001, USP10’s roles in regulating diverse cellular processes and different diseases, especially cancers, have been widely investigated [9,51]. In brief, USP10 is a double-edged sword in affecting human tumorigenesis due to the complex cellular contexts. Therefore, given the oncogenic role of USP10, the development of more specific USP10 inhibitors could be a potential and promising strategy for cancer treatment. To date, only Compounds 3 and 9 of the HBX19818 analogs are more specific for USP10, while other inhibitors, including spautin-1 (the first to be developed for USP10), are not specific for USP10 and have at least two targets [61,105]. Bearing this notion in mind, it is necessary and urgent to screen or develop much more specific USP10 inhibitors. In addition to directly targeting USP10, alternative approaches also need to be considered, such as the inhibition of USP10 by regulating its upstream regulators, including USP13, estrogen, and microRNAs (as summarized in Table 2). On the other hand, due to their nature and low side effects, using natural agents such as quercetin and Traditional Chinese Medicine as USP10 inhibitors could be a safe and effective strategy for cancer therapy. Although some upstream regulators and substrates of USP10 have been identified, in-depth investigations of both the underlying mechanisms of USP10-mediated tumorigenesis and the development of corresponding drugs are urgently needed. Mounting evidence indicates that enzymes also exert multiple functions in an enzymatic-activity-independent fashion, such as EZH2. Therefore, notably, we also need to pay more attention to exploring the additional role of USP10 outside its deubiquitinase activity. In light of the double-sided role of USP10 in tumorigenesis, it would be fascinating to explore the physiological role of USP10 in the progression of different tumors by using conditional knockout (KO) or knock-in mouse models. Looking forward, cancer-type-specific USP10 animal models will accelerate and improve the development of specific USP10 inhibitors. We believe that inhibitors of USP10 with the best specificity and efficacy will be developed and used in clinical trials for the therapy of different cancers in the near future. 

## Figures and Tables

**Table 1:** Summary of the identified deubiquitination substrates for USP10.

Substrate	Role USP10 Plays	Mechanism Summary
Yki	USP10 deubiquitinates Yki and stabilizes Yki protein.	USP10 binds to Yki and promotes Yki deubiquitination and stabilization through the proteasome ubiquitination pathway, while Usp10 may regulate human YAP activity [48].
RPS3	USP10 recovers ribosomal subunits from translation blockages.	RPS3 is monoubiquitinated by Hel2p E3 ligase, which is regulated by the interaction between Hel2p and UBP3 [11].
AR	USP10 is a cofactor that binds to AR and stimulates the androgen response of the target promoter	USP10 regulates the activity of AR. USP10 releases AR in the cytosol and enhances the nuclear entry and transcriptional activity of AR, thus affecting the AR signaling pathway [49].
H2A	USP10 affects AR-mediated gene expression.	USP10 directly deubiquitinates H2A [32].
PCNA	USPs regulates the stability of DNA polymerase ETA.	USPs can regulate/inhibit oxidative stress or uV-induced ubiquitination of proliferating cell nuclear antigen (PCNA) [50].
p53	USP10 has been identified as a regulator of p53.	Under non-stress conditions, USP10 releases p53 in the cytoplasm, thus countering MDM2’s action and allowing nuclear re-entry. During DNA damage, USP10 accumulates in the nucleus, is phosphorylated by ATM, and deubiquitinates p53 in the nucleus [27,51].
p62	USP10 inhibits apoptosis.	USP10 interacts with P62, and this interaction enhances p62-dependent ubiquitination protein aggregation and aggregator formation [52].
AMPKα	USP10 mediates AMPKα decoupling to regulate autophagy.	USP10 activity promotes the LKB1 phosphorylation of AMPKα at Thr172. USP10 stabilizes AMPKα by inhibiting AMPKα ubiquitination in HCC, which results in the inhibition of AKT and mTOR activation [15].
Beclin-1	USP10 also regulates beclin-1, a key promoter of autophagy.	Beclin-1 stabilizes USP13, which in turn deubiquitinates and stabilizes USP10, leading to an increase in beclin-1 levels and activity. Finally, USP10 deubiquitinated and activated the autophagy-promoting kinase AMPK [53].
NICD1	USP10 affects vascular morphology.	USP10 regulates Notch signaling during angiogenic spouting by interacting with and stabilizing the NOTCH1 intracellular domain (NICD1) in endothelial cells. Notch signaling is important in determining the germinating behavior of endothelial cells [54].
G3BP	USP10 regulates deubiquitination activity and membrane transport between endoplasmic reticulum and Golgi apparatus by binding to G3BP.	Stress granules (SGs) are dynamic RNA–protein complexes located in the cytoplasm that rapidly form under stress and disperse when normal conditions return. The formation of SGs is dependent on the SH3-domain-binding protein (G3BP) of RAS-Gap. USP10 binds to the G3BP protein to form the USP10–G3BP1 complex, which is required for the deubiquitination of RPS2, RPS3, and RPS10. Thus, the modified 40S subunit is saved from degradation [55,56,57].
SIRT6	SP10 inhibits SIRT6 ubiquitination, thereby protecting it from proteasomal degradation.	USP10 antagonizes the transcriptional activation of the c-Myc oncogene through SIRT6 and TP53, thereby inhibiting cell-cycle progression, cancer cell growth, and tumor formation [25].
PTEN	USP10 inhibits the growth and invasion of lung cancer cells by the upregulation of PTEN.	USP10 stabilizes PTEN by inhibiting PTEN ubiquitination in HCC [58].
MSH2	The USP10–MSH2 pathway regulates the DNA-damage response.	USP10 interacts with and stabilizes MutS homolog 2 (MSH2) in lung cancer cells [32].
mTOR	USP10 inhibits hepatocellular carcinoma (HCC) growth in vivo by inhibiting the mTOR signaling pathway.	USP10 interacts with RNF168 and TOP2α to inhibit ubiquitination and chromatin binding of TOP2α [59].
MEK	USP10 induced MEK1 activation and reduced apoptosis.	USP10 stabilizes the ITCH E3 ligase’s binding with MEK1, leading to the degradation of MEK1 and reduced plasma membrane localization of PTEN [60].
FLT3-ITD	USP10 regulates FLT3–ITD stabilization.	USP10 selectively deactivates and stabilizes mutated FLT3–ITD, resulting in FLT3–ITD-promoting carcinogenic cell proliferation [61].
SYK	USP10 inhibition is a novel approach to inhibiting splenic tyrosine kinase (SYK) and impeding its role in AML pathology, including tumorigenic FFLT3-positive AML.	USP10 forms a complex with FLT3–ITD and physically binds to SYK, stabilizing the levels of both proteins by deubiquitinating USP10. USP10 can directly interact with SYK [62].
TIA1	USP10 regulates the content of TIA1-/Tau-positive stress particles.	TIA1-/Tau-positive stress particles were severely weakened by USP10 depletion [63].
CFTR	USP10 promotes the endocytic cycle of CFTR by deubiquitination	USP10 mediated the desorption of cystic fibrosis transmembrane conduction regulator (CFTR) in early endosomes, thus enhancing the endocytosis and recirculation of CFTR. It also directly interacts with CFTR and deubiquitinates CFTR, resulting in increased expression of CFTR on the cell surface [39,64].
Nexin 3	The expression of USP10 increases the SNX3 protein level.	USP10 increases SNX3 expression by deubiquitination and reducing proteasomal degradation, both of which promote ENaC’s export to the plasma membrane through the secretory pathway [65].
HDAC6	USP10 is a deubquitination enzyme (DUB) for HDAC6.	Two full-length catalytic domains of HDAC6, N-terminal DAC1, and central DAC2 bind to the C-terminal of USP10 [32,58].
CD36	USP10 regulates CD36 expression and promotes foam cell formation.	CD36 is a substrate of USP10; upon their interaction, USP10 stabilizes CD36 through deubiquitination [16].
Smad4	USP10 promotes metastasis of liver cancer.	USP10 directly interacts with Smad4 and reverses the polymerization of the ubiquitin chain linked to proteolytic Lys48, resulting in its stabilization and the activation of TGF-β signaling, which promotes HCC metastasis [46].
αv integrin	USP10 is important for myofibroblast development.	USP10 deubiquitinated β1 and β5 integrins, particularly αvβ5 and αvβ1, but not αvβ3, leading to cell-surface integrin accumulation and subsequent local TGF-β activation [66].
LC3B	USP10 deubiquitinates LC3B and increases the LC3B level and autophagic activity.	USP10 silencing reduces lc3B-I and LC3B-II forms of LC3B by increasing ubiquitination and proteasomal degradation [67].
NLRP7	USP10-mediated NLRP7 deubiquitination promotes tumor progression and tumor-associated macrophage polarization in colorectal cancer.	NLRP7 interacts with USP10 to catalyze deubiquitination in colorectal cancer (CRC) cells. K379 is an important lysine receptor site [68].
P14ARF	USP10 deubiquitinates P14ARF and improves its stability.	USP10 transcription induced by c-Myc improved the stability of the p14ARF protein [69].
DnaK	DnaK binds to USP10 and affects p53-dependent anticancer functions.	USP10 deubiquitinates p53, regulating the DNA-damage response; DnaK interacts with USP10, inhibiting its deubiquitination activity [34].
KLF4	USP10 deubiquitinates KLF4 and regulates KLF4 protein levels.	Activation of USP10–KLF4–TIMP3 signaling axis inhibits the occurrence of lung cancer [70].

**Table 2:** Summary of the identified forms of regulation for USP10.

Upstream Regulator	Mechanism Summary	Ref#
AMPKα	AMPKα increases its activity by mediating phosphorylation of Ser76 at the USP10 N-terminus.	[15]
ATM	ATM mediates phosphorylation of USP10 at Thr42 and Ser337 and causes USP10’s migration into the nucleus.	[27]
TRAF4	TRAF4 and p53 competitively bind to USP10 and inhibit usP10-mediated p53 deubiquitination.	[73]
AKT	Co-stimulation by BCR and TLR1/2 initiates Akt-dependent phosphorylation of T674 in the USP10 NLS domain.	[72]
USP13/beclin-1	When USP10 and USP13 interact with beclin-1, the deubiquitination activity of USP10 can be increased.	[53]
MiR-138	MiR-138 binds to a conserved region of USP10’s 3′-UTR and inhibits the accumulation of USP10 mRNA and protein expression level.	[31]
MicroRNA-191	MicroRNA-191 binds to the 3′-untranslated region of USP10 mRNA, reducing USP10 protein levels.	[28]
MiR-34a-5p	MiR-34a-5p binds to the 3′-untranslated region of USP10 and reduces the expression of USP10.	[79]
G3BP	Direct binding of G3BP to USP10 inhibits its ability to decompose ubiquitin chains.	[56]
HTLV-1 Tax	The central region of Tax interacts with amino acids 727–798 in USP10, inhibiting the activity of USP10.	[78]
Daam1	Daam1 negatively regulates USP10’s DUB activity.	[80]
Estrogen	Estrogen induces p53 degradation by regulating USP10 activity.	[81]
FOXO4	Overexpressed FOXO4 inhibits USP10 transcription and protein expression by binding to the bases 1771–1776 in the promoter region TSS of USP10.	[82]

## Data Availability

Not applicable.
